# *In vitro* anticoccidial activity of nanoencapsulated bromelain against *Eimeria* spp. oocysts isolated from goats in Kenya

**DOI:** 10.14202/vetworld.2022.397-402

**Published:** 2022-02-22

**Authors:** Ahmota Romain Daiba, John Maina Kagira, Maina Ngotho, James Kimotho, Naomi Maina

**Affiliations:** 1 Department of Molecular Biology and Biotechnology, Pan-African University of Institute of Basic Science, Technology and Innovation, Nairobi, Kenya; 2 Department of Animal Sciences, Jomo Kenyatta University of Agriculture and Technology, Nairobi, Kenya; 3 Department of Clinical Studies, University of Nairobi, Nairobi, Kenya; 4 Innovation and Technology Transfer Division, Kenya Medical Research Institute, Nairobi, Kenya; 5 Department of Biochemistry, Jomo Kenyatta University of Agriculture and Technology, Nairobi, Kenya

**Keywords:** anticoccidial activity, bromelain, chitosan, coccidia, goat, nanoencapsulation

## Abstract

**Background and Aim:**

The emergence of drug-resistant strains of *Eimeria* spp. calls for the development of novel anticoccidial drugs. Plant extracts provide a possible natural source for such drugs. This study aimed to investigate the *in vitro* anticoccidial activity of encapsulated bromelain (EB) in chitosan nanocarriers on *Eimeria* spp. oocysts isolated from goats kept by farmers in Kenya.

**Materials and Methods:**

Bromelain was extracted from the peel of ripe pineapples using standard methods. *Eimeria* spp. oocysts were isolated from the feces of goats using a flotation method. The inhibition of sporulation was assayed after exposing the oocysts to solutions of EB, non-EB (NEB), and diclazuril (positive control) at concentrations between 4 mg/mL and 0.125 mg/mL for 48 h. The oocysts were examined under a microscope (40x) to determine the effects of the drugs on the sporulation process. The percentage of sporulation inhibition was calculated after 48 h and the inhibition concentration 50% (IC_50_) was determined by probit analysis.

**Results:**

Bromelain manifested anticoccidial activity through the inhibition of the sporulation of coccidia oocysts. EB achieved inhibition with a lower dose compared with NEB. The IC_50_ values of diclazuril, EB, and NEB were 0.078 mg/mL, 0.225 mg/mL, and 0.575 mg/mL, respectively. There were significant differences (p<0.01) between the IC_50_ of EB and NEB compared with the standard treatment drug.

**Conclusion:**

This preliminary study showed that EB has anticoccidial activity supporting further evaluation at an *in vivo* level to develop a novel drug for the management of coccidiosis in goats.

## Introduction

Worldwide, intestinal coccidiosis is one of the most important parasitic diseases of small ruminants [1]. The disease is caused by protozoan parasites belonging to the genera *Eimeria* spp. and *Isospora* spp. that develop in the small and large intestines and have devastating effects on younger animals [2,3]. The most common *Eimeria* species in goats in Kenya and Africa in general are *Eimeria ninakohlyakimovae*, *Eimeria hirci*, *Eimeria caprina*, *Eimeria christenseni*, *Eimeria jolchijevi*, *Eimeria apsheronica*, and *Eimeria arloingi* [4-7]. Coccidiosis losses in small ruminants are due to clinical and subclinical infections often associated with poor weight gain, reduced production, and increased mortality in younger stock [1,8]. In studies conducted in East Africa, coccidiosis appears to be a leading cause of mortality among small ruminants. It can compound the occurrence of other parasitic and infectious diseases, such as pneumonia and helminthosis [5,7,9].

Coccidiosis is mainly managed through anticoccidials and anticoccidiostats; these can be either administered orally or through feed and water. The current anticoccidial drugs include toltrazuril, diclazuril, decoquinate, amprolium, and sulfonamide [10-12]. However, the overuse and misuse of these drugs have led to the emergence of drug-resistant strains of *Eimeria* spp. [13]. This has stimulated the development of novel drugs with plant extracts being considered as potential sustainable alternatives. Herbal extracts, such as *Curcuma longa*, *Artemisia absinthium*, *Saussurea lappa*, *Ageratum conyzoides*, *Olea europaea*, *Ruta pinnata*, and *Trachyspermum ammi*, have been shown to have antiparasitic activity and to enhance the immune system and growth performance, thereby helping the host to overcome coccidiosis infection [14,15].

One plant that has been shown to exert appreciable levels of antiparasitic activity is the pineapple (*Ananas comosus*). The pineapple is a common tropical fruit grown in various countries, including Kenya [16]. Bromelain is the main compound extracted from pineapples and has been characterized as a mixture of cysteine proteases found in the tissues of plants from the Bromeliaceae family. The commercially available proteolytic enzymes extracted from pineapple are fruit and stem bromelain [17]. Besides its anthelmintic properties, bromelain possesses a wide range of therapeutic properties, such as antibacterial and anti-inflammatory effects and the ability to enhance drug absorption [18-20]. However, its effects against intestinal protozoan infection, such as coccidia, have not been investigated. One of the main challenges of bromelain is to maintain stability within the gastrointestinal system of animals. Recent studies have shown that the encapsulation of bromelain by chitosan can stabilize and maintain the activity of bromelain throughout the gut [16].

This study aimed to evaluate the *in vitro* anticoccidial activity of encapsulated bromelain (EB) against coccidia affecting goats and developing a novel drug for the management of coccidiosis in goats.

## Materials and Methods

### Ethical approval

Since no handling procedure was performed on the animals, approval from the Institutional Animal Ethics Committee to conduct the study was not required.

### Study period and location

The study was conducted for a period of 5 months (February 2021 to June 2021). The study was carried out at Jomo Kenyatta University of Agriculture Technology, Juja, Kiambu County, Kenya located at latitude 1°05 S and longitude 37°00 E. It lies at an altitude of 1525 m above sea level and receives an annual rainfall of 850 mm with an average temperature of 18.7°C [16].

### Extraction and chitosan encapsulation of bromelain

Bromelain was extracted from the stem and peels of pineapple (*A. comosus*) which was purchased from the local market in Juja Sub-County, Kenya. The enzyme was extracted using the procedure described by Hunduza *et al*. [20]. Briefly, fresh ripe pineapples were cut into small pieces and then ground in a blender in sodium acetate buffer, pH 7.4. The resultant crude extract was sieved and then precipitated by adding 40% ammonium sulfate salt. The extracted bromelain was purified using a 12 kDa dialysis membrane (Thermo Scientific, USA). The protein concentration was measured using a Nanodrop spectrophotometer (polymerase chain reaction max, Lambda, VacuTec, Germany). The ionic gelation method was used to encapsulate bromelain into chitosan by mixing equal volumes (30 mL each) of extracted bromelain (4 mg/mL), which was mixed with 1% sodium tripolyphosphate (STPP, Dentex Industries Ltd, Kenya) using a rotary mixer. Then, 12 mL of the bromelain-STPP mixture was added to 20 mL of 1% chitosan (Sigma-Aldrich, USA) under vigorous magnetic stirring and then sonicated for 45 min. The resultant suspension was centrifuged at 15,000 rpm for 45 min and the obtained pellet was washed with distilled water before freeze-drying. The aliquots of bromelain-loaded chitosan nanocarrier pellet were frozen at –60°C and dried in a freeze-dryer (MRC, Model FDL-10N-50-BA, Israel). The successful conjugation of bromelain to the chitosan nanoparticles was confirmed by Fourier-transform infrared spectroscopy (Shimadzu 8400, Japan) [20]. Protease activity was performed following the method described by Devakate *et al*. [21]. Briefly, 1 mL of bromelain was incubated with 5 mL of casein (as a substrate) for 10 min at 37°C. Trichloroacetic acid 98% extra pure (Loba Chimie Pvt. Ltd., India) was added and incubated for 30 min at 37°C. After the mixture was filtered through filter paper (Whatman No.1, GE Healthcare, Chicago, USA), 5 mL of sodium carbonate and 1 mL of Folin–Ciocalteu reagent (Loba Chimie Pvt. Ltd.) were added and incubated for 30 min at 37°C. The absorbance of the clear supernatant was measured at 660 nm.

### Isolation and collection of *Eimeria* spp. oocysts

Oocyst samples of *Eimeria* spp. were isolated from fresh field fecal samples collected from goats kept by farmers in Juja Sub-County, Kenya. The harvesting of oocysts was a flotation technique using saturated salt (NaCl) solution [22,23]. Briefly, a 5 g sample of feces was weighed and mixed in a mortar. The mixture was dissolved in 50 mL of tap water and filtered through a sieve with a 105 μm aperture. The filtrate was centrifuged for 8 min at 1000 rpm. The supernatant was discarded and the sediment was suspended in a solution of 40% NaCl solution (specific gravity 1:2) and allowed to stand for 10 min to allow coarse fecal material to sink, minimizing the chance of trapping oocysts. The suspension was then centrifuged at 400 rpm for 6 min. Then, approximately 5 mL was aspirated from the top and the oocysts suspension was further washed by centrifugation (at 1000 rpm for 8 min) twice in distilled water. The supernatant was discarded and the oocysts were resuspended from the sediment in 2.5% (w/v) potassium dichromate [K_2_Cr_2_O_7,_ (Bio-Chem, France)]. solution and then used directly to perform anticoccidial assays.

### *In vitro* anticoccidial tests

The *in vitro* sporulation inhibition assay was evaluated using the method described by Odden *et al*. [12]. Briefly, 15 mL Eppendorf tubes (Thermo Scientific™ Nunc™ 15mL) containing a total volume of 2 mL of each concentration of the encapsulated and non-EB (NEB) (4, 2, 1, 0.5, 0.25, and 0.125 mg/mL) were prepared. The tubes were then inoculated with an equal volume of unsporulated oocyst suspension in K_2_Cr_2_O_7_ and incubated at 28°C for 48 h. For comparison, diclazuril (Vetranal^®^, Sigma-Aldrich) was used as the positive control. A suspension of oocysts in K_2_Cr_2_O_7_ alone was used as the negative control. A serial dilution series of chitosan solution (4, 2, 1, 0.5, 0.25, and 0.125 mg/mL) was used as the control. At the end of incubation, the effect of the test EB on the oocysts sporulation was examined under a microscope at 40× at both 48 and 72 h. The numbers of sporulated and non-sporulated oocysts were counted and the percentage inhibition was estimated by counting the number of unsporulated oocysts from a total of 100 oocysts. Three replicates were measured for each concentration. The sporulation inhibition percentage was calculated as described by Cedric *et al*. [24];


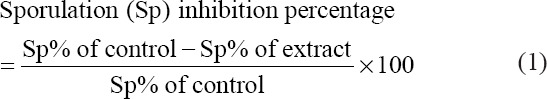


### Statistical analysis

The obtained data were entered into and analyzed using the Statistical Package for the Social Sciences software version 28.0 (IBM Corp., NY, USA). The concentration at which inhibition concentration 50% (IC_50_) of sporulation occurred was determined using the regression line of probit in accordance with the log_10_ of the extract concentration. The mean percentages at different concentrations and ratios were compared using paired sample t-tests, with p<0.05 considered to indicate statistical significance.

## Results

### Bromelain concentration and activity

The protein content of crude and purified bromelain was 7.11 mg/mL and 2.80 mg/mL, respectively. The protease activity of purified bromelain was 0.0930 units/mL, whereas that of the EB was 0.0070 units/mL.

### *In vitro* anticoccidial effect

**Figure-1:**
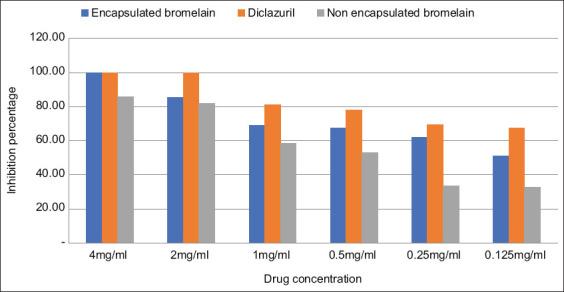
Effect of encapsulated bromelain, diclazuril and non-encapsulated bromelain on % sporulation inhibition after 48 h.

**Figure-2:**
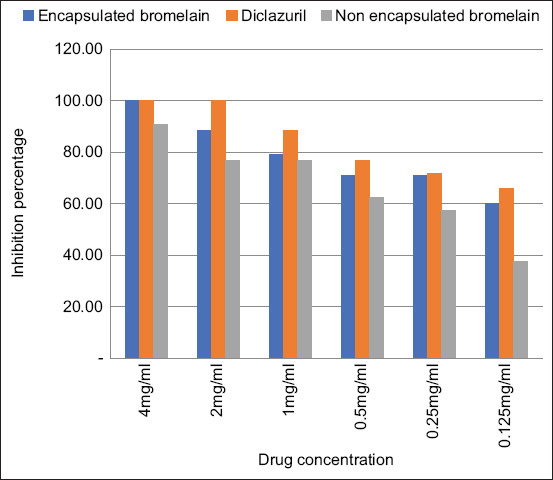
Effect of encapsulated bromelain, diclazuril, and non-encapsulated bromelain on % sporulation inhibition after 72 h.

The *in vitro* anticoccidial activity of the chitosan-EB against coccidial oocysts was examined after 48 h and 72 h which is summarized in Figures-1 and 2, respectively. The results showed that at all concentration levels, the EB inhibited sporulation of coccidia oocysts. The lowest concentration (0.125 mg/mL) inhibited sporulation of more than 50% of the oocysts after incubation for 48 h (Figure-1) and by 60% after 72 h. However, sporulation increased in the negative controls (Figure-2). The results also showed that chitosan alone did not affect sporulation inhibition, even at the highest concentration tested (4 mg/mL).

The EB had significantly higher (p<0.05) anticoccidial activity than unencapsulated bromelain. In contrast, diclazuril had significantly higher (p<0.05) anticoccidial activity than EB. The IC_50_ values for diclazuril, EB, and plain bromelain were 0.078, 0.225, and 0.575 mg/mL, respectively (Table-1).

**Table 1:** IC_50_ values of encapsulated bromelain, diclazuril, and non-encapsulated bromelain on oocysts sporulation (with 95% confidence limits for concentration) (mg/mL).

Drug	Lower boundary (mg/mL)	Upper boundary (mg/mL)	Average (mg/mL)
Encapsulated bromelain	0.193	0.257	0.225^a^
Diclazuril	0.001	0.211	0.078^b^
Non-encapsulated bromelain	0.478	0.673	0.575^c^

For the same column, values carrying the same superscript letter are not significantly different at p≥0.05 (t-test), IC_50_=Inhibition concentration 50%

### Oocyst damage caused by EB

**Figure-3:**
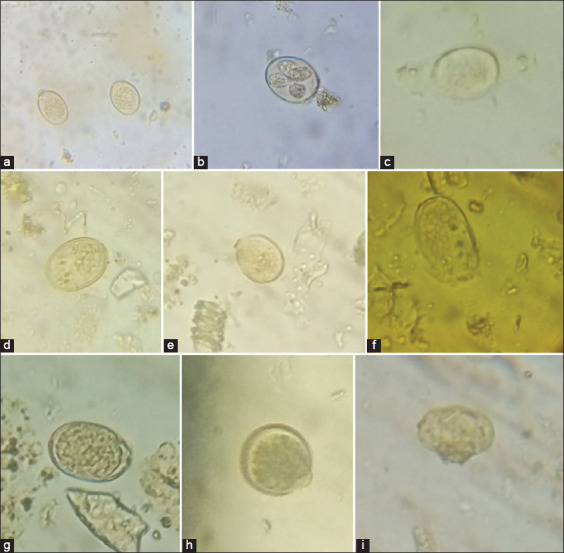
Changes observed after exposure of *Eimeria* oocysts to encapsulated bromelain (under the microscope 40×): (a) Normal coccidian oocysts; (b) normal sporulation from control group; (c-i) abnormal and unsporulated oocysts in EB.

After coccidia were exposed to EB, the changes observed included weakening of the shell, bursting of the oocyst at the weakest point, and the destruction of the central cytoplasmic mass oocyst (Figure-3). Normal sporulation was observed in oocysts exposed to chitosan and water as negative controls.

## Discussion

The use of plant extracts to develop new drugs against coccidia is the subject of a number of studies. This is because the current conventional drugs face problems, such as the emergence of drug-resistant coccidial species and consumer concern regarding the presence of drug residues in milk and meat [25,26]. The present study aimed to test the anticoccidial efficacy of bromelain nanoencapsulated by chitosan on *Eimeria* spp. oocysts isolated from the feces of goats kept by farmers in Kenya. As reported in the previous studies, the inhibition of sporulation and the damage to oocysts were used as the criteria for assessing anticoccidial properties [24,27].

Our study showed that the encapsulation of low concentrations (0.125 mg/mL) of bromelain inhibited sporulation of more than 50% of *Eimeria* spp. oocysts. The IC_50_ for EB was higher than that of pure bromelain, showing that the encapsulation process could increase the inhibition activity of bromelain. This confirms that EB has anticoccidial activity and can be developed further as drugs for coccidiosis. The previous studies have shown that extracts from plants, including *A. conyzoides* (*Asteraceae*), olive pulp (*O. europaea* L. var. *Chemlal)*, and Canary rue (*R. pinnata*), have activity against *Eimeria* spp. oocyst sporulation [15,28,29]. Molan *et al*. [30] also observed the inhibition of sporulation *in vitro* when avian *Eimeria tenella*, *Eimeria maxima*, and *Eimeria acervulina* were exposed to aqueous extracts of pine bark (*Pinus radiata*). The sporulation inhibition was 28-84% by 500 μg/mL of *P. radiata* extract [30], whereas our study recorded more than 50% sporulation inhibition with 125 μg/mL bromelain. In addition, Hur *et al*. [31] studied the effects of condensed tannin-containing plants on natural coccidian infection in goats. They observed that goats fed pine needles and oak leaves in combination with Lucerne chaff resulted in a reduction in oocysts.

Our study showed that bromelain nanoencapsulated by chitosan affected the coccidia sporulation process; the extract caused severe damage to the morphology of coccidian oocysts. As observed from their activity against the eggs of helminths, the bromelain extracts may inhibit sporulation by inactivating the endogenous enzymes responsible for the sporulation process [24,32]. Another study showed that the plant extracts could penetrate both layers of the oocyst shell and cause a loss of intracellular components, which would result in the destruction and softening of the central cytoplasmic mass [33]. In the present study, the EB would have affected the shell wall of oocysts, softening it and causing damage to the central cytoplasmic mass (sporont), as evidenced by the observation of abnormal sporocysts in oocysts exposed to higher concentrations.

This study has demonstrated the value of EB as an anticoccidial agent by the inhibition of sporulation of *Eimeria* spp. oocysts, even at the lowest concentration tested (0.124 mg/mL). This provides preliminary indicative data that the molecule may be suitable for development into a drug to control protozoal parasites.

## Conclusion

EB has anticoccidial activity against *Eimeria* spp. oocysts isolated from goats (51% of sporulation inhibition at 0.125 mg/ml and 100% at 4 mg/ml). Further studies should be conducted to determine the *in vivo* efficacy of EB for the treatment of coccidiosis in goats. This will inform ongoing studies geared toward the development of EB as a novel drug that can be used to manage coccidial diseases affecting livestock.

## Authors’ Contributions

ARD, JMK, MN, JK and NM: Involved in the conception of the research idea. ARD and JMK: Planned the study design. ARD, JK, and MN: Performed sample (feces) collection and laboratory work. ARD: Drafted the manuscript. JMK, MN, JK, and NM: Corrected the manuscript. All authors read and approved the final manuscript.
